# Metastases of soft tissue sarcoma to the liver: A Historical Cohort Study from a Hospital‐based Cancer Registry

**DOI:** 10.1002/cam4.3304

**Published:** 2020-07-10

**Authors:** Masanori Okamoto, Masatake Matsuoka, Tamotsu Soma, Ryuta Arai, Hidenori Kato, Toru Harabayashi, Hirohumi Adachi, Toshiki Shinohara, Tamotsu Sagawa, Noriaki Nishiyama, Toshikazu Nambu, Wataru Sakai, Hiroaki Suzuki, Hiroyuki Kato, Hiroaki Hiraga

**Affiliations:** ^1^ Sarcoma Center National Hospital Organization Hokkaido Cancer Center Sapporo Japan; ^2^ Department of Musculoskeletal Oncology National Hospital Organization Hokkaido Cancer Center Sapporo Japan; ^3^ Department of Orthopaedic Surgery Shinshu University School of Medicine Nagano Japan; ^4^ Department of Gynecologic Oncology National Hospital Organization Hokkaido Cancer Center Sapporo Japan; ^5^ Department of Urology National Hospital Organization Hokkaido Cancer Center Sapporo Japan; ^6^ Department of Thoracic Surgery National Hospital Organization Hokkaido Cancer Center Sapporo Japan; ^7^ Department of Gastrointestinal Surgery National Hospital Organization Hokkaido Cancer Center Sapporo Japan; ^8^ Department of Medical Oncology National Hospital Organization Hokkaido Cancer Center Sapporo Japan; ^9^ Department of Radiation Oncology National Hospital Organization Hokkaido Cancer Center Sapporo Japan; ^10^ Department of Diagnostic Radiology National Hospital Organization Hokkaido Cancer Center Sapporo Japan; ^11^ Department of Pathology National Hospital Organization Hokkaido Cancer Center Sapporo Japan

**Keywords:** leiomyosarcoma, liver neoplasms, neoplasm metastasis, retroperitoneal neoplasm, soft tissue sarcoma

## Abstract

**Background:**

Hepatic metastasis of soft tissue sarcoma is rare compared to lung metastasis, and the literature is scarce. We examined the risk of hepatic metastasis according to the site of occurrence and histological type.

**Methods:**

From a Hospital‐based Cancer Registry, 658 patients registered between 2007 and 2017 with soft tissue sarcomas were evaluated. The exclusion criteria were gastrointestinal stromal tumors, tumors of unknown origin, and follow‐up periods of less than 1 month. SPSS 25 was used for statistical analysis.

**Results:**

The risk of hepatic metastasis was significantly higher in the retroperitoneum (HR, 5.981; 95% CI, 2.793‐12.808) and leiomyosarcoma (HR, 4.303; 95% CI, 1.782‐10.390). Multivariate analysis showed that the risk of hepatic metastasis as first distant metastasis was high in leiomyosarcoma (HR, 4.546; 95% CI, 2.275‐9.086) and retroperitoneal onset (HR, 4.588; 95% CI, 2.280‐9.231). The 2‐year survival rate after hepatic metastasis was 21.7%.

**Conclusions:**

The onset of hepatic metastasis indicates a poor prognosis. However, hepatic metastasis from retroperitoneal sarcoma and leiomyosarcoma may be the first distant metastasis in some cases. For retroperitoneal sarcoma and leiomyosarcoma, additional screening for hepatic metastasis such as contrast CT should be considered during staging and follow‐up after treatment.

## Graphical abstract

Following the analysis of 658 cases of soft tissue sarcomas excluding gastrointestinal stromal tumors registered in the Hospital‐based Cancer Registry from 2007 to 2017, we found that the percentage of retroperitoneal sarcomas and leiomyosarcomas causing hepatic metastasis was high at first visit, as the first distant metastasis, and throughout the entire course of the disease. For retroperitoneal sarcomas and leiomyosarcomas, additional screening for hepatic metastases should be considered during staging and postoperative follow‐up.

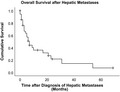

## 1 INTRODUCTION

Soft tissue sarcoma is a malignant tumor that arises in non‐epithelial extraskeletal tissues, excluding the reticuloendothelial system, glia, and supportive tissues of various parenchymal organs.^1^ The lungs are the most common site of occurrence and comprise 80% of the first site of metastasis from soft tissue sarcomas.^2^ However, among the many forms of soft tissue sarcomas, some unusual patterns of metastatic spread have been reported in the literature, such as extrapulmonary metastasis of myxoid liposarcoma ^3^ and brain metastasis of alveolar soft part sarcoma.^4^ Thus, according to the National Comprehensive Cancer Network Guidelines, non‐contrast CT is necessary for accurate staging. Abdominal and pelvis CT are recommended for angiosarcoma, leiomyosarcoma, myxoid liposarcoma, and epithelioid sarcoma. An MRI of the total spine is also recommended for myxoid liposarcoma, as well as an MRI of the central nervous system for alveolar soft part sarcoma and angiosarcoma. The European Society for Medical Oncology Clinical Practice Guideline also states that local MRI and chest CT are often performed for postoperative follow‐up.^5^ Furthermore, the 3‐year survival rate after surgical resection for lung metastasis from soft tissue sarcoma is reported as 54%, and complete resection is recommended if complete resection of the metastasis is possible.^6^

While there are many reports on lung metastases, studies on hepatic metastases from soft tissue sarcomas are scarce.^7^, ^8^ In recent years, several reports have shown results of local therapy for hepatic metastasis of soft tissue sarcoma.^9^, ^10^, ^11^, ^12^ Although these studies describe soft tissue sarcomas in the retroperitoneum and peritoneal cavity as being prone to hepatic metastases, many of these findings are based on gastrointestinal stromal tumor and few have reported on other types of soft tissue sarcomas. Gastrointestinal stromal tumor is classified as a soft tissue sarcoma according to the WHO classification^13^; however, treatment with tyrosine kinase inhibitors was introduced in the early aughts and has dramatically improved the clinical outcome of gastrointestinal stromal tumors.^14^ Therefore, when analyzing the clinical results of soft tissue sarcomas, it is currently a common practice to analyze gastrointestinal stromal tumors and other soft tissue sarcomas separately.

The purpose of this study is to clarify the difference in the incidence of hepatic metastasis in soft tissue sarcomas based on sites of occurrence and histological types, exclusive of gastrointestinal stromal tumors.

## 2 MATERIALS AND METHODS

This study was approved by the Institutional Review Board of our hospital. The data used in this study were obtained from the Hospital‐based Cancer Registry.

A total of 24 552 malignant tumor cases were collected in the Hospital‐based Cancer Registry from January 1 of 2007 to December 31 of 2017. The inclusion criteria comprised of patients with soft tissue sarcoma, and 687 cases (2.8%) were included. Exclusion criteria were as follows: gastrointestinal stromal tumors, tumors of unknown origin, and follow‐up periods of less than 1 month. These patients were analyzed to establish risk factors for hepatic metastases presenting various clinical features of soft tissue sarcoma.

Based on the evaluation of radiology reports by a radiologist, the diagnosis of hepatic metastasis was determined by either a new appearance or gradually enlarging nodular shadow on CT and MRI imaging tests performed at our hospital. The direct invasion of primary tumor into the liver was not classified as hepatic metastasis. Potential risk factors for hepatic metastases included age at first visit, gender, histological type, site of occurrence for primary tumor, size, presence or absence of distant metastasis (lung, liver, and other sites of involvement) and time of onset, follow‐up period, outcome at final follow‐up, 2‐year and 5‐year survival rates from first visit, and the 1‐year and 2‐year survival rates after indication of hepatic metastasis were evaluated. The site of occurrence of the primary tumor was divided into the following five groups: extremity, body wall, retroperitoneal, thoracic and peritoneal, and head and neck. According to a report by Jaques et al,^7^ visceral sarcomas were classified as thoracic and peritoneal. Histological type was divided into nine groups based on the WHO classification, which included adipocytic tumors, fibroblastic/myofibroblastic tumors, the so‐called fibrohistiocytic tumors, smooth muscle tumors (leiomyosarcoma), skeletal muscle tumors, vascular tumors of soft tissue, nerve sheath tumors, tumors of uncertain differentiation, and undifferentiated/unclassified sarcomas.^13^

IBM SPSS Statistics for Windows, Version 25.0 was used for statistical analysis. Survival curves were generated by the Kaplan‐Meier method. For multivariate analysis, the hazard ratio and 95% confidence interval were calculated using the Cox proportional hazards model. *P* < .05 was considered significant.

## 3 RESULTS

Of 687 soft tissue sarcomas that were registered, 13 cases were gastrointestinal stromal tumors, 1 case was of an unknown primary site, and 15 cases were censored at less than 1 month after registration. A total of 658 cases remained after these exclusions, comprising of 323 males and 335 females with a median age at first visit of 65.0 years (range, 1‐96). The median follow‐up was 35.0 months (range, 0‐135). There were 128 patients who were diagnosed with sarcoma following primary tumor resection at another hospital, and these patients were referred to our hospital immediately after resection for additional treatment. In 32 patients who had inadequate resection at another hospital, an additional wide resection was performed at our hospital. A total of 434 surgical resections of the primary lesion were performed at our hospital, and 96 patients did not undergo surgery. Chemotherapy was performed in 156 patients, and radiotherapy was performed in 146 patients (Table 1). For the primary surgery at our hospital, preoperative radiotherapy was performed if the tumor was in close proximity to important organs such as neurovascular bundles, and postoperative radiotherapy was performed if the postoperative margin was either a close or positive margin. Histological diagnoses and their respective location of tumor are outlined in Table 2.

**TABLE 1:** Summary of treatment strategies for primary tumors

	Total	Chemotherapy	Radiotherapy
Resected at another hospital	128	37	23
Rested at our hospital	434	92	92
Did not undergo surgery	96	27	49
Total	658	156	146

**TABLE 2:** Diagnosis and tumor location

	Extremity	Body wall	Retro‐peritoneal	Thoracic and peritoneal	Head and neck	Total
Adipocytic tumors	136	23	41	9	0	209
Fibroblastic/myofibroblastic tumors	58	28	0	2	5	94
So‐called fibrohistiocytic tumors	0	1	0	1	0	2
Smooth muscle tumors	20	7	16	25	1	69
Skeletal muscle tumors	8	1	0	2	1	12
Vascular tumors of soft tissue	2	3	0	2	5	12
Nerve sheath tumors	14	6	3	2	6	31
Tumors of uncertain differentiation	36	11	1	9	2	59
Undifferentiated/unclassified sarcomas	102	44	12	9	3	170
Total	376	124	74	61	23	658

**Figure 1:**
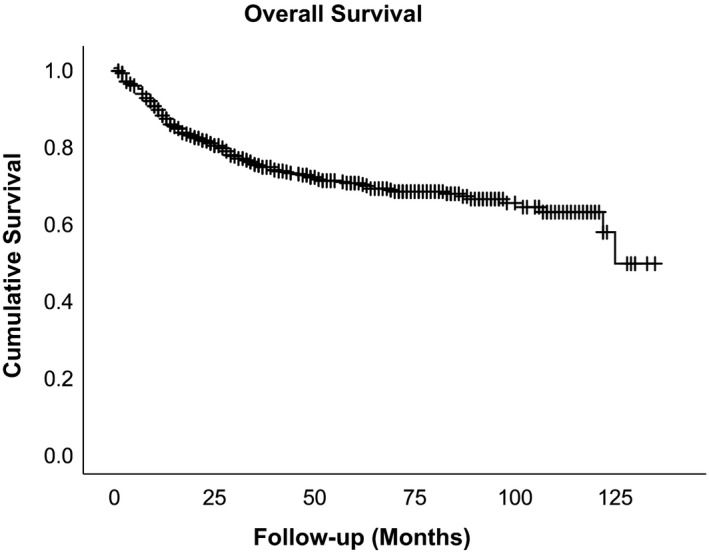
Kaplan‐Meier survival curves of all cases

Adipocytic tumors were the most common histological type (209 cases), followed by undifferentiated/unclassified sarcomas (170 cases) and leiomyosarcoma (69 cases). The site of occurrence was most common in the extremities (376 cases), followed by the body wall (124 cases) and retroperitoneum (74 cases). Details on histological type are described in Table S1. The 2‐year cumulative overall survival rate was 80.3% (95% confidence interval 77.2‐83.4), and the 5‐year cumulative overall survival rate was 70.0% (95% confidence interval 66.1‐74.0) (Figure 1). At first examination, the size of primary tumors was less than 10 cm in 240 cases and more than 10 cm in 276 cases. We did not observe a significant difference in the risk of hepatic metastasis by age, gender, or size of the primary tumor at initial visit.

Table 3 shows the presence of hepatic metastasis according to the location of tumor. The retroperitoneum accounted for the highest percentage of hepatic metastasis at initial examination (6.8%), the highest incidence of hepatic metastasis at first metastasis detected as the first relapse (10.8%), and hepatic metastasis at the last day of follow‐up of this study (17.6%). Compared to the extremities which account for the largest number of cases, retroperitoneal hepatic metastasis at first metastasis demonstrated a hazard ratio of 5.793 and a 95% confidence interval of 2.164‐15.09. The incidence of hepatic metastasis at the last day of follow‐up in this study demonstrated a hazard ratio of 5.981 and a 95% confidence interval of 2.793‐12.808.

**TABLE 3:** Presence of hepatic metastases according to location and time of onset

	At initial presentation	At first metastasis detected as the first relapse		At last day of follow‐up of the present study	
			Hazard ratio (95% CI)^a^		Hazard ratio (95% CI)^a^
Extremity	3/376 (0.8%)	8/376 (2.1%)	NA	14/376 (3.7%)	NA
Body wall	0/124	1/124 (0.8%)	0.404 (0.051‐3.234)	3/124 (2.4%)	0.663 (0.190‐2.306)
Retroperitoneal	5/74 (6.8%)	8/74 (10.8%)	5.793 (2.164‐15.509)	13/ 74 (17.6%)	5.981 (2.793‐12.80)
Thoracic and peritoneal	2/61 (3.3%)	3/61 (4.9%)	3.016 (0.793‐11.472)	6/ 61 (9.8%)	3.355 (1.283‐8.775)
Head and neck	0/23	0/23	NA	0/23	NA
Total	10/658 (1.5%)	20/658 (3.0%)	NA	36/658 (5.5%)	NA

Abbreviations: CI, Confidence interval; NA, Not assessed.

^a^ Univariate cox proportional hazard model comparing each site of occurrence to the extremities.

The presence of hepatic metastasis according to histological type is outlined in Table 4. Leiomyosarcoma exhibited the highest percentage of hepatic metastasis at initial presentation (8.7%), the highest incidence of hepatic metastasis at first metastasis (11.6%), and hepatic metastasis at the time of the last day of follow‐up in this study (18.8%). Compared to undifferentiated/unclassified sarcomas, leiomyosarcoma demonstrated a hazard ratio of 4.085 for hepatic metastasis at first metastasis and a 95% confidence interval of 1.794‐9.302. The incidence of hepatic metastases at the last day of follow‐up in this study demonstrated a hazard ratio of 4.303 and a 95% confidence interval of 1.782‐10.390.

**TABLE 4:** Presence of hepatic metastasis according to histological type and time of onset

	At initial presentation	At first metastasis detected as the first relapse		At last day of follow‐up in the present study	
			Hazard ratio (95% CI)^a^		Hazard ratio (95% CI)^a^
Adipocytic tumors	1/209 (0.5%)	3/209 (1.4%)	0.381 (0.125‐1.166)	5/209 (2.4%)	0.462 (0.151‐1.414)
Fibroblastic/Myofibroblastic tumors	1/94 (1.1%)	1/94 (1.1%)	0.289 (0.050‐1.671)	4/94 (4.3%)	0.829 (0.249‐2.762)
So‐called fibrohistiocytic tumors	0/2	0/2	NA	0/2	NA
Smooth muscle tumors	6/69 (8.7%)	8/69 (11.6%)	4.085 (1.794‐9.302)	13/69 (18.8%)	4.303 (1.782‐10.390)
Skeletal muscle tumors	1/12 (8.3%)	1/12 (8.3%)	3.014 (0.521‐17.426)	2/12 (16.7%)	4.301 (0.910‐20.328)
Vascular tumors of soft tissue	0/12	0/12	NA	1/12 (8.3%)	2.120 (0.265‐16.988)
Nerve sheath tumors	0/31	2/31 (6.5%)	1.813 (0.491‐6.688)	2/31 (6.5%)	1.354 (0.287‐6.391)
Tumors of uncertain differentiation	0/59	1/59 (1.7%)	0.575 (0.100‐3.321)	1/59 (1.7%)	0.354 (0.044‐2.828)
Undifferentiated/Unclassified sarcomas	1/170 (0.6%)	4/170 (2.4%)	NA	8/170 (4.7%)	NA
Total	10/658 (1.5%)	20/658 (3.0%)	NA	36/658 (5.5%)	NA

Abbreviations: CI, Confidence interval; NA, Not assessed.

^a^ Univariate cox proportional hazard model comparing each site of occurrence to undifferentiated/unclassified sarcomas

In terms of multivariate analysis, retroperitoneal sarcoma and leiomyosarcoma were significantly associated with the risk of hepatic metastasis at first metastasis and the development of hepatic metastasis at the last day of follow‐up in this study (Table 5). Eight of 16 retroperitoneal leiomyosarcomas (50%) developed hepatic metastases.

**TABLE 5:** Multivariate cox proportional hazard model for hepatic metastasis

	At first metastasis detected as the first relapse			At last day of follow‐up in the present study		
	Hazard Ratio	95% CI	*P* value	Hazard Ratio	95% CI	*P* value
Leiomyosarcoma	5.589	2.223‐14.053	<.001	4.546	2.275‐9.086	<.001
Retroperitoneal	4.505	1.797‐11.296	.001	4.588	2.280‐9.231	<.001
Thoracic and peritoneal	―	―	―	―	―	―

Abbreviation: CI, Confidence interval.

**Figure 2:**
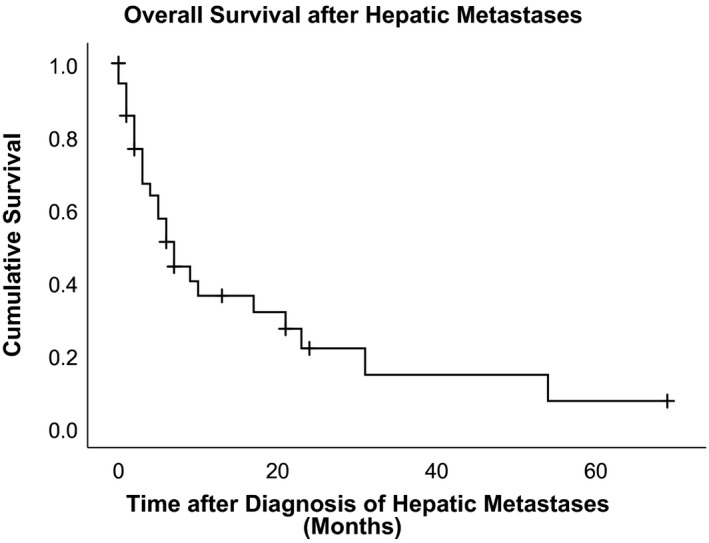
Kaplan‐Meier survival curves after indication of hepatic metastasis

The 1‐year cumulative overall survival rate was 36.1% (95% confidence interval 18.7‐53.6) after indication of hepatic metastasis, and the 2‐year cumulative overall survival rate was 21.7% (95% confidence interval 5.0‐38.4) (Figure 2).

## 4 DISCUSSION

In this study, we analyzed the difference in incidence of hepatic metastases based on the site of occurrence and histological type for 658 soft tissue sarcomas registered in a hospital‐based cancer registry from 2007 to 2017. As a result, retroperitoneal sarcoma and leiomyosarcoma were significantly associated with the risk of hepatic metastasis at first examination, hepatic metastasis as the first distant metastasis, and hepatic metastasis during the entire course of disease.

The “anatomical‐mechanical” and “seed and soil” hypotheses have long been well‐known theories to determine the metastatic destination of malignant tumors. The anatomical‐mechanical theory was proposed by Ewing et al in 1919.^15^, ^16^ The theory postulates that the direction of blood flow determines the organ specificity of metastasis and has been confirmed both clinically and in basic research, particularly for gastrointestinal cancer.^17^, ^18^ Gastrointestinal cancer is thought to metastasize to the liver hematogenously via the portal vein. Because the blood flow from the retroperitoneum also passes through the portal vein, our results that suggest retroperitoneal sarcoma is associated with the risk of hepatic metastasis was consistent with this theory. The seed and soil theory was proposed by Paget et al in 1889 and postulates that the establishment of metastasis requires a microenvironment suitable for the growth of cancer cells.^19^ Furthermore, in recent years, it has been clarified that exosomes derived from primary lesions are taken up by cells to which they are transferred and form a niche suitable for metastasis.^20^ Since the intrinsic nature of the tumor determines the organ specificity of the metastatic destination, the occurrence of metastasis is specific to its histology, as in skin metastasis of leiomyosarcoma^21^ and lymphatic metastasis of hemangiosarcoma, fetal rhabdomyosarcoma, and epithelioid sarcoma.^22^ In our study, leiomyosarcoma was associated with the risk of hepatic metastasis. It has been long reported that the liver and lungs are common sites of metastasis for leiomyosarcoma.^23^, ^24^ However, prior to the late 1990s when KIT staining became widely available, most gastrointestinal stromal tumors were diagnosed with leiomyosarcoma based on histological criteria, and older reports on leiomyosarcoma mainly consisted of patients with gastrointestinal stromal tumors.^25^ Fletcher et al have reported that leiomyosarcoma is the most common sarcoma that causes skin metastasis, in addition to soft tissue and bone metastases.^13^ On the other hand, even if leiomyosarcoma and gastrointestinal stromal tumor are correctly diagnosed by KIT staining, there are reports that hepatic metastasis from leiomyosarcoma remains common,^26^, ^27^ and no consensus has been reached. The results of this study also identified the risk of leiomyosarcoma and hepatic metastasis.

The effectiveness of lung metastasis resection for soft tissue sarcoma is widely recognized,^6^ and surgical treatment such as surgery for single hepatic metastasis has been reported to be as comparatively effective as lung metastasis resection.^12^, ^28^ The response rate of chemotherapy for hepatic metastasis has been reported to be low,^24^ and the prognosis after indication of hepatic metastasis in this study was also poor. Assuming that an early detection of single hepatic metastasis leads to an improvement in prognosis, patients with retroperitoneal sarcoma and leiomyosarcoma should undergo screening for hepatic metastasis during the staging process or follow‐up after treatment. Non‐contrast CT, as recommended by guidelines, may underestimate the presence of hepatic metastases.^29^ Contrast CT or MRI should therefore be considered for the screening of hepatic metastases.

There are several limitations to this study. First, this was a retrospective cohort study (historical cohort study) using a hospital‐based cancer registry at a single institution. Second, in soft tissue sarcomas that arise in the limb, screening for hepatic metastasis such as abdominal CT is not routinely performed and may underestimate the risk of hepatic metastasis. Routine abdominal imaging examinations were not performed in this study. Even if a patient underwent abdominal CT imaging, most were plain CT scans. Lastly, our hospital has only performed one surgical operation for hepatic metastasis, and analyses of treatment outcomes and cost‐effectiveness for the surgical treatment of hepatic metastasis of retroperitoneal sarcoma and leiomyosarcoma were not performed in this study and warrants further investigation.

## 5 CONCLUSIONS

Hepatic metastases of soft tissue sarcomas are relatively rare. The occurrence of hepatic metastasis indicates a poor prognosis. There was no hepatic metastasis from the head and neck. However, retroperitoneal sarcoma and leiomyosarcoma may cause early hepatic metastasis, and hepatic metastasis may be the first distant metastasis in some cases. For retroperitoneal sarcoma and leiomyosarcoma, additional screening for hepatic metastasis such as contrast CT should be considered during staging and follow‐up after treatment.

## 6 DECLARATION OF INTEREST

None.

## 7 CONFLICT OF INTEREST STATEMENT

None declared.

## AUTHORS’ CONTRIBUTIONS

**Masanori Okamoto:** Conceived and designed the study, curated the data, formally analyzed and interpreted the data, and wrote the original draft. **Masatake Matsuoka:** Curated the data, formally analyzed and interpreted the data, and reviewed the article. **Tamotsu Soma:** Curated the data, formally analyzed and interpreted the data, and reviewed the article. **Ryuta Arai:** Curated the data, formally analyzed and interpreted the data, and reviewed the article. **Hidenori Kato:** Curated the data, formally analyzed and interpreted the data, and reviewed the article. **Toru Harabayashi:** Curated the data, formally analyzed and interpreted the data, and reviewed the article. **Hirohumi Adachi:** Curated the data, formally analyzed and interpreted the data, and reviewed the article. **Toshiki Shinohara:** Curated the data, formally analyzed and interpreted the data, and reviewed the article. **Tamotsu Sagawa:** Curated the data, formally analyzed and interpreted the data, and reviewed the article. **Noriaki Nishiyama:** Curated the data, formally analyzed and interpreted the data, and reviewed the article. **Toshikazu Nambu:** Curated the data, formally analyzed and interpreted the data, and reviewed the article. **Wataru Sakai:** Curated the data, formally analyzed and interpreted the data, and reviewed the article. **Hiroaki Suzuki:** Curated the data, formally analyzed and interpreted the data, and reviewed the article. **Hiroyuki Kato:** Reviewed and edited the article. **Hiroaki Hiraga:** Conceived and designed the study and reviewed and edited the article.

## Supporting information

Table S1

## Data Availability

The data that support the findings of this study are available on request from the corresponding author. The data are not publicly available due to privacy or ethical restrictions.
