# Supplementary material for: Metastases of soft tissue sarcoma to the liver: A Historical Cohort Study from a Hospital‐based Cancer Registry
Source: Cancer Med. 2020 Jul 10;9(17):6159–65. doi: 10.1002/cam4.3304 (PMC7476817; doi:10.1002/cam4.3304)
Supplement: Supplementary file 1 — Table S1 [file CAM4-9-6159-s001.docx]

**Supplementary Table 1**　 **Details on Diagnosis and Tumor Site**

|  | Extremity | Body wall | Retroperitoneal | Thoracic and peritoneal | Head and neck | Total |
| --- | --- | --- | --- | --- | --- | --- |
| **Adipocytic tumors** |  |  |  |  |  |  |
| Atypical lipomatous tumor | 79 | 12 | 0 | 1 | 0 | 92 |
| Dedifferentiated liposarcoma | 13 | 6 | 19 | 5 | 0 | 43 |
| Myxoid liposarcoma | 39 | 3 | 2 | 0 | 0 | 44 |
| Pleomorphic liposarcoma | 5 | 1 | 1 | 0 | 0 | 7 |
| Well differentiated liposarcoma | 0 | 0 | 18 | 2 | 0 | 20 |
| Liposarcoma, not otherwise specified | 0 | 1 | 2 | 1 | 0 | 4 |
| **Fibroblastic/Myofibroblastic tumors** |  |  |  |  |  |  |
| Dermatofibrosarcoma protuberans | 7 | 9 | 0 | 0 | 3 | 19 |
| Fibrosarcomatous dermatofibrosarcoma protuberans | 1 | 3 | 0 | 0 | 0 | 4 |
| Low-grade myofibroblastic sarcoma | 1 | 0 | 0 | 0 | 1 | 2 |
| Myxofibrosarcoma | 40 | 11 | 1 | 0 | 1 | 53 |
| Sclerosing epithelioid fibrosarcoma | 1 | 1 | 0 | 0 | 0 | 2 |
| Solitary fibrous tumor, Malignant | 5 | 1 | 0 | 2 | 0 | 8 |
| Infantile fibrosarcoma | 1 | 0 | 0 | 0 | 0 | 1 |
| Fibrosarcoma | 2 | 3 | 0 | 0 | 0 | 5 |
| **So-called fibrohistiocytic tumors** |  |  |  |  |  |  |
| Tenosynovial giant cell tumor, malignant | 0 | 1 | 0 | 0 | 0 | 1 |
| Giant cell tumor of soft tissue | 0 | 0 | 0 | 1 | 0 | 1 |
| **Smooth muscle tumors** |  |  |  |  |  |  |
| Leiomyosarcoma | 20 | 7 | 16 | 25 | 1 | 69 |
| **Skeletal muscle tumors** |  |  |  |  |  |  |
| Rhabdomyosarcoma | 2 | 0 | 0 | 0 | 0 | 2 |
| Embryonal rhabdomyosarcoma | 1 | 0 | 0 | 2 | 1 | 4 |
| Pleomorphic rhabdomyosarcoma | 4 | 1 | 0 | 0 | 0 | 5 |
| Alveolar rhabdomyosarcoma | 1 | 0 | 0 | 0 | 0 | 1 |
| **Vascular tumors of soft tissue** |  |  |  |  |  |  |
| Epithelioid hemangioendothelioma | 0 | 0 | 0 | 1 | 0 | 1 |
| Angiosarcoma of soft tissue | 2 | 3 | 0 | 1 | 5 | 11 |
| **Nerve sheath tumors** |  |  |  |  |  |  |
| Malignant peripheral nerve sheath tumor | 13 | 6 | 3 | 2 | 5 | 29 |
| Malignant perineurinoma | 0 | 0 | 0 | 0 | 1 | 1 |
| Malignant Triton tumor | 1 | 0 | 0 | 0 | 0 | 1 |
| **Tumors of uncertain differentiation** |  |  |  |  |  |  |
| Synovial sarcoma | 12 | 7 | 0 | 0 | 0 | 19 |
| Synovial sarcoma, biphasic | 6 | 1 | 0 | 0 | 0 | 7 |
| Epithelioid sarcoma | 3 | 0 | 0 | 1 | 0 | 4 |
| Alveolar soft part sarcoma | 7 | 0 | 0 | 0 | 1 | 8 |
| Clear cell sarcoma of soft tissue | 4 | 1 | 0 | 1 | 0 | 6 |
| Extraskeletal Ewing sarcoma | 3 | 0 | 1 | 5 | 1 | 10 |
| CIC-rearranged round cell sarcoma | 0 | 2 | 0 | 1 | 0 | 3 |
| Extra-renal rhabdoid tumor | 1 | 0 | 0 | 0 | 0 | 1 |
| Intimal sarcoma | 0 | 0 | 0 | 1 | 0 | 1 |
| **Undifferentiated/Unclassified sarcomas** |  |  |  |  |  |  |
| Undifferentiated pleomorphic sarcoma | 86 | 35 | 6 | 2 | 2 | 131 |
| Undifferentiated sarcoma | 8 | 6 | 2 | 6 | 0 | 22 |
| Undifferentiated spindle cell sarcoma | 8 | 3 | 3 | 1 | 1 | 16 |
| **Total** | 376 | 124 | 74 | 61 | 23 | 658 |

‘‘continued’’
